# A ubiquitylation site in Cockayne syndrome B required for repair of oxidative DNA damage, but not for transcription-coupled nucleotide excision repair

**DOI:** 10.1093/nar/gkw216

**Published:** 2016-04-07

**Authors:** Michael Ranes, Stefan Boeing, Yuming Wang, Franziska Wienholz, Hervé Menoni, Jane Walker, Vesela Encheva, Probir Chakravarty, Pierre-Olivier Mari, Aengus Stewart, Giuseppina Giglia-Mari, Ambrosius P. Snijders, Wim Vermeulen, Jesper Q. Svejstrup

**Affiliations:** 1 Mechanisms of Transcription Laboratory, The Francis Crick Institute, Clare Hall Laboratories, South Mimms EN6 3LD, UK; 2 Department of Genetics, Cancer Genomics Netherlands, Erasmus MC, P.O. Box 2040, 3000 CA Rotterdam, Netherlands; 3 Protein Analysis and Proteomics Laboratory, The Francis Crick Institute, Clare Hall Laboratories, South Mimms EN6 3LD, UK; 4 Bioinformatics & Biostatistics Laboratory, The Francis Crick Institute, 44 Lincoln's Inn Fields, London WC2A 3LY, UK; 5 Institut de Pharmacologie et de Biologie Structurale, Centre National de la Recherche Scientifique, F-31077 Toulouse, France

## Abstract

Cockayne syndrome B (CSB), best known for its role in transcription-coupled nucleotide excision repair (TC-NER), contains a ubiquitin-binding domain (UBD), but the functional connection between protein ubiquitylation and this UBD remains unclear. Here, we show that CSB is regulated via site-specific ubiquitylation. Mass spectrometry analysis of CSB identified lysine (K) 991 as a ubiquitylation site. Intriguingly, mutation of this residue (K991R) does not affect CSB's catalytic activity or protein stability, but greatly affects genome stability, even in the absence of induced DNA damage. Moreover, cells expressing CSB K991R are sensitive to oxidative DNA damage, but proficient for TC-NER. K991 becomes ubiquitylated upon oxidative DNA damage, and while CSB K991R is recruited normally to such damage, it fails to dissociate in a timely manner, suggesting a requirement for K991 ubiquitylation in CSB activation. Interestingly, deletion of CSB's UBD gives rise to oxidative damage sensitivity as well, while CSB ΔUBD and CSB K991R affects expression of overlapping groups of genes, further indicating a functional connection. Together, these results shed new light on the regulation of CSB, with K991R representing an important separation-of-function-mutation in this multi-functional protein.

## INTRODUCTION

Eukaryotic cells employ multiple pathways to maintain genome integrity (1). For example, nucleotide excision repair (NER) removes bulky DNA lesions such as those resulting from UV-irradiation, while base excision repair (BER) repairs damage to bases such as that generated by oxidation. Cockayne Syndrome B protein (CSB) plays a role in both these pathways. Indeed, cells carrying mutations in CSB are sensitive to UV-irradiation and display a dramatic delay in the recovery of transcription after DNA damage (2–4). CSB mutation also increases the sensitivity to various oxidative DNA damaging agents (5–7). Interestingly, CSB, a translocase of the SWI/SNF-family of DNA-dependent ATPases (8,9), appears to be particularly important for the repair of transcription-perturbing DNA lesions, so-called transcription-coupled repair. During transcription-coupled NER (TC-NER), CSB is essential for establishing functional repair complexes at damage-stalled RNAPII (10,11). The biochemical basis for the involvement of CSB in BER is less clear, but recent data suggest that the role is direct and that the activity of CSB is transcription-dependent (12,13).

Although CSB has been intensively studied for many years and its importance in disease development and the DNA damage response is well established (reviewed by (14,15), many questions regarding the regulation of this multi-functional protein remain unanswered. Recently, we identified a functionally important ubiquitin-binding domain at the C-terminus of the CSB protein (16), and several other connections between CSB and protein ubiquitylation have been reported. For example, CSB is ubiquitylated by a ubiquitin ligase complex containing the CSA protein, which can result in proteasome-mediated proteolysis (17,18). More recently, it was found that UVSSA, which itself contains a ubiquitin-binding domain, stabilizes CSB by delivering the ubiquitin protease USP7 to the TC-NER complex, which may represent a critical regulatory mechanism of this process (19–21).

To further investigate the connection between ubiquitylation and CSB function, we mapped sites of ubiquitylation in the CSB protein. Here, we show that one of these sites, lysine 991, is important for genome stability, but not for TC-NER. Instead, ubiquitylation at this site is important for the role of CSB in the response to oxidative DNA damage.

## MATERIALS AND METHODS

### Protein purification and ATPase assay

8xHIS-FLAG-CSB constructs were transfected into 293T cells using calcium phosphate and overexpressed proteins were purified in three steps on Ni^2+^-NTA agarose beads (Qiagen) followed by ANTI-FLAG M2-agarose beads (Sigma) and a final step again on Ni^2+^-NTA agarose beads. Details are available on request. Measurements of ATPase activity were performed in 15 μl reactions in 10 mM Tris–HCl (pH 7.5), 50 mM NaCl, 0.5 mM MgCl_2_, 0.5 mM DTT, 100 μM cold ATP, 2.5 μM of [α-^32^P] ATP (800 Ci/mmol), 80 μg BSA, and in the presence of 100 ng CSB for 60 min at 37°C and the reaction was stopped with 1.5 μl of 0.5 M EDTA. Where indicated the reaction was supplemented with 250 ng double-stranded λ-DNA (NEB). Four microliters of the reaction was spotted onto CEL300PEI-cellulose plates (Machery-Nagel) to separate ADP and ATP by thin-layer chromatography in 1 M formic acid, 0.3 M LiCl, and results were visualised by Phosphorimager exposure and autoradiography film exposure (GE Healthcare).

### Multiple sequence alignments

Protein sequence alignments of various CSB homologs using the ClustalW2 web-based tool (22), with default parameters and completed manually. The NCBI (predicted) protein sequences used: human (NP000115), mouse (XP484360), dog (XP534944.2), chicken (XP_004942197.1), zebrafish (XP_688972.2), opossum (XP_001366076.1) and puffer fish (Uniprot ID H3DGI8).

### Survival and recovery of RNA synthesis after UV-irradiation

UV sensitivity of the CSB inducible cell lines were determined by clonogenic survival assay as previously described (23). Cells were fixed and stained using published methods (24). To measure RNA synthesis after UV-irradiation, cells were pulse labelled with ^3^H-uridine as described elsewhere (25).

### MultiDsk experiments

MultiDsk resin was prepared as previously described (26). Pull-down of ubiquitylated proteins was preformed by incubating 15 μl MultiDsk resin with 500 μg WCE for 4 h at 4°C. The beads were washed 4 times with 1 ml lysis buffer for 5 min each. Afterward, the beads were resuspended in 60 μl 1× SDS-loading buffer and boiled for 10 min at 95°C. Fifteen microliters of sample was used immunoblotting.

### Alkaline comet assay

Comet assay was performed according to Trevigen comet assay kit protocol for alkaline comet assay of adherent cells. Samples were visualized using Axioskop epifluorescence microscope with 40× magnification. Comet parameters were analysed automatically with image acquisition and analysis software package Comet IV (Perceptive Instruments).

### Cell viability assay

CellTiter-Glo Luminescent cell viability assay (Promega) was used to measure the sensitivity of cells to different DNA damaging agents. The assay was performed in 96-well plates, black/clear bottom (BD Biosciences). Two thousand cells/well were seeded and incubated overnight. DNA damage treatments were assayed through internal triplicates by culturing the cells for 3 days in the presence of increasing concentrations of the damaging agent. Samples were processed according to manufactures guidelines (Promega) and luminescence was measured using the Pherastar FS (BMG LabTecch) 96-well plate reader. The average of the triplicates for each dose were calculated, the values from the untreated samples were used to normalize and to calculate cell viability after treatment with the different DNA damaging agent concentrations. The cell viability of the untreated samples was set to 100%.

\begin{equation*} {\rm Cell}\;{\rm viability}\;(\% ) = ({\rm values}\;{\rm after}\;{\rm treatment}/{\rm untreated}\;{\rm value}) \times 100 \end{equation*}

### Time-lapse microscopy

GFP-tagged CSB expression was induced by supplementing the growth media with 5–10 ng/ml doxycycline. Mitotic duration was performed in 12-well plates. An hour prior to imaging, complete DMEM media was changed to CO_2_-independent media without phenol red supplemented with 5–10 ng/ml doxycycline. Phase contrast images of cells were then acquired at 37°C using a Zeiss Axio observer Z1 microscope controlled by SimplePCI software (Hamamatsu), equipped with Ocra 03GO1 CCD camera (Hamamatsu) and a Plan-Apochromat 10×/0.45 DIC H objective. The live imaging was performed over 48 h with multiple recording positions per well with 5 min interval between image acquisitions. This represents ±600 image acquisitions per position over the 48 h imaging period. Images were processed using ImageJ software and analysed using mitotic duration plugin. The image frames between mitotic cell rounding (start of mitosis) and appearance of the cleavage furrow (transition from mitosis to cytokinesis) were counted and the number of frames were multiplied by 5 min, hereby resulting in the mitotic duration in minutes for that particular cell. Approximately 50 events were counted for each cell line used.

### Cell cycle analysis

Asynchronous cells expressing doxycycline induced GFP-CSB were harvested by trypsinization and cell pellets were fixed with 70% ethanol. Standard FACS analysis based on DNA content was performed by resuspending cells staining solution (25 μg/ml propidium iodide), 10 μg/ml RNaseA (DNase free, PBS) and incubating them for 1 h at 37°C in the dark. Data was acquired using the BD FACS Calibur flow cytometer and data was processed using FlowJo computer software.

### Live-cell confocal laser-scanning microscopy for inducing oxidative and UV-C damage

The expression of GFP-tagged CSB proteins was induced by supplementing the growth media with 5–10 ng/ml doxycycline. Oxidative DNA damage induction was described previously (12). In short, Ro 19-8022 photosensitizer (5 μM), a gift of F. Hoffmann-La Roche, Ltd, was added 10 min before starting the experiment and activated by a 405-nm laser (20% laser power), for 3.6 s in a square of 2 × 2 μm using a SP5 confocal microscope (Leica) with a 63× objective lens. GFP-CSB accumulation was measured over time and relative fluorescence signals were measured, background corrected and normalized for the average pre-damage signals.

Protein mobility upon oxidative DNA damage was analysed by FRAP, as described previously (27) with some modifications. Briefly, two ROIs of 2 × 2 μm in and outside of the damage were monitored every 0.46 s with a 488 nm-laser of a SP5 confocal microscope (Leica) with a 63× objective lens. GFP-CSB was bleached at the ROIs for 1.38 s at maximum intensity of the laser. Recovery of the fluorescent molecules was recorded as relative fluorescence. FRAP data were normalized to the average pre-bleached fluorescence. All data were normalized to the average pre-damaged relative fluorescence after removal of the background signal. For each experiment and cell type, 15 cells were measured.

### Gene expression analysis

Cells were cultured in DMEM containing 10% (vol/vol) FBS [Tet system-approved FBS for inducible cell lines] at 37°C with 5% CO_2_. To achieve equal expression of the different CSB versions, doxycycline (10 ng/ml for CS1AN, WT and K991R; 5 ng/ml for ΔUBD) was added to cells 60 h before total RNA was extracted. Total RNA was extracted with an RNeasy Mini Kit (Qiagen), according to the manufacturer's instructions. The integrity of the RNA was tested on a denaturing agarose gel. RNA quality and quantity were also assessed with a Nanodrop spectrophotometer (Thermo Fisher Scientific). Double-stranded cDNA was syntheized from 10 μg of total RNA, using the cDNA synthesis kit according to the NimbleGen user protocol. Up to 1 μg of double-stranded cDNA was labeled with Cy3 and hybridized on a NimbleGen 12 × 125K Human Expression Array (2007-09-12_HG18_opt_expr), followed by washing and drying according to the manufacturer's instructions (NimbleGen-Roche). Arrays were scanned with a NimbleGen MS200 microarray scanning system, and data acquisition was performed with NimbleScan according to the manufacturer's recommendations. Data are deposited in the Gene Expression Omnibus database (GSE77680).

## RESULTS

**Figure 1.**
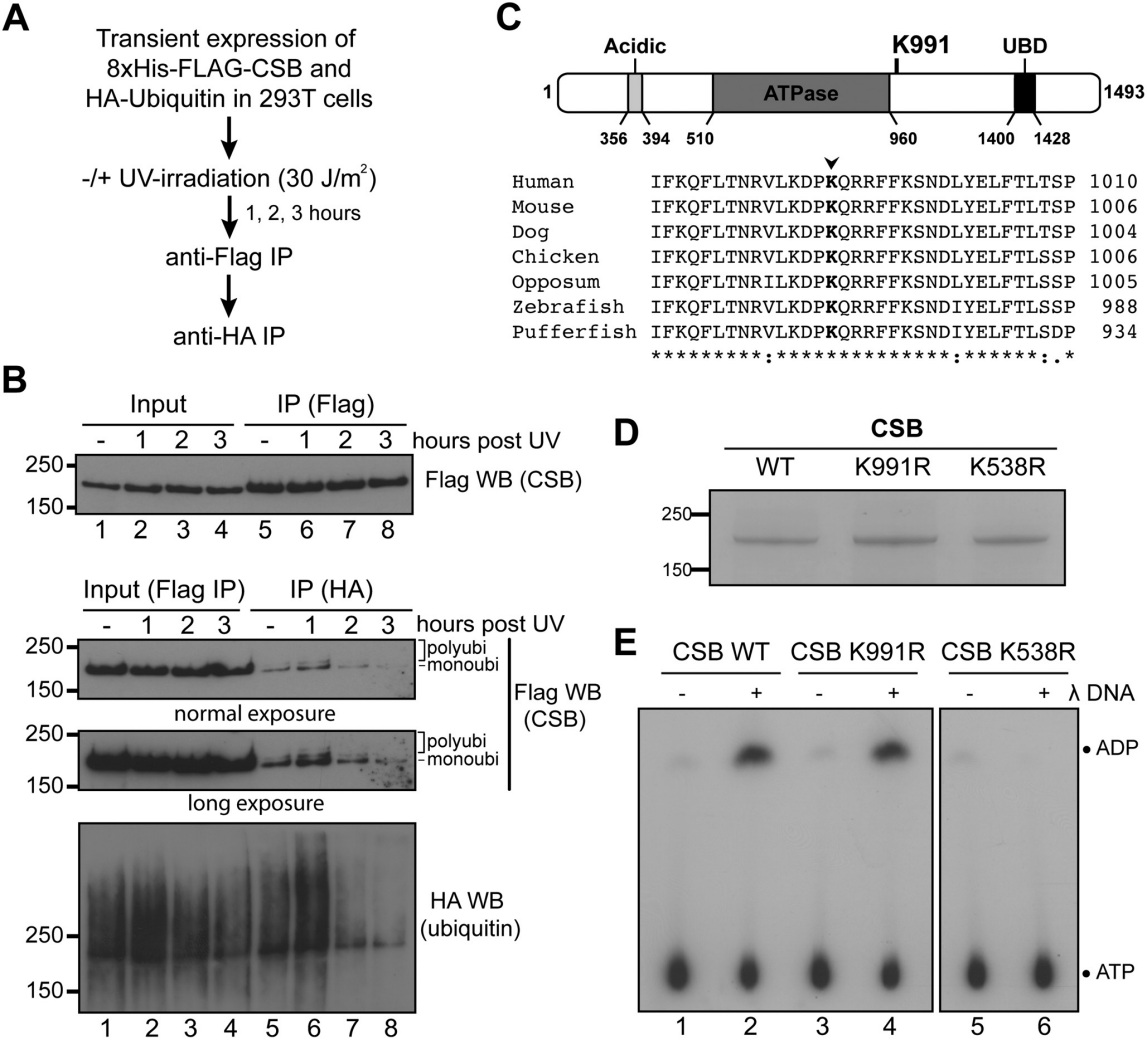
Identification of CSB ubiquitylation sites. (**A**) Outline of the experimental approach. **B**) Upper: 8xHIS/FLAG-CSB was affinity purified from whole cell extracts using anti-FLAG resin. Lower: ubiquitylated CSB was affinity purified from the FLAG elution fractions using anti-HA resin. (**C**) Upper, Lysine 991 in relation to known CSB domains. Lower, Multiple sequence alignments of CSB vertebrate orthologues around lysine 991 (red). (**D**) Coomassie-stained gel of affinity purified CSB proteins. (**E**) ATPase assay with the indicated CSB proteins in the absence (−) or presence (+) of DNA.

In order to investigate CSB ubiquitylation, we transiently co-expressed 8xHis/FLAG-tagged CSB and HA-tagged ubiquitin in 293T cells and then employed a dual immuno-purification protocol to isolate the modified protein (Figure 1A). Using this protocol, CSB indeed appeared to be ubiquitylated in response to UV-irradiation (Figure 1B), with the detection of what appeared to predominantly be mono-ubiquitylated CSB, but also some multi- or poly-ubiquitylated forms of CSB (lower three panels, lane 6), which decreased at later time-points (lanes 7 and 8). Interestingly, however, we also detected significant amounts of ubiquitylated CSB at steady state, even in the absence of DNA damage (Figure 1B, lane 5). Together, these results suggest that CSB is ubiquitylated both before and after UV-induced DNA damage.

### Identification of novel ubiquitylation sites on CSB

After large-scale immuno-precipitation of ubiquitylated CSB using the same procedure, the protein was subjected to mass spectrometry analysis. In agreement with previous studies (28,29), lysines (K) 1392 and 1457 were identified as CSB ubiquitylation sites both before and after DNA damage (Supplementary Figure S1A). Neither of these lysines are conserved in higher eukaryotes (Supplementary Figure S1B), and we previously found that the function of the region encompassing these sites can be provided by a heterologous UBD-containing protein fragment derived from yeast Rad23 (16). We therefore reasoned that these C-terminal ubiquitylation sites were less likely to be of regulatory importance and did not pursue them further. However, we also detected ubiquitylation at K991 (Supplementary Figure S1C), in a UV-independent fashion. K991 is located just outside the core CSB ATPase domain (Figure 1C, upper). This, combined with the high degree of conservation of this amino acid in CSB orthologues (Figure 1C, lower), spurred us to investigate whether K991 ubiquitylation might be important.

K991 was mutated to an arginine (CSB K991R). Wild-type CSB (WT), CSB K991R and the ATPase mutant CSB K538R were then expressed in HEK293T cells and purified to near homogeneity (Figure 1D). CSB K991R had levels of DNA-dependent ATPase activity that were similar to those of WT, whereas—as expected—no significant activity was observed for the ATPase mutant K538R (Figure 1E). We conclude that mutation of lysine 991 to arginine does not alter the basic catalytic activity of CSB.

### Genome instability phenotypes of CSB K991R mutation

We now went on to examine the importance of the CSB K991 ubiquitylation site *in vivo*. Initially, the immortalised CSB-deficient patient cell line CS1ANsv was used to generate cell lines that constitutively expressed 8xHIS/FLAG-tagged versions of CSB WT and CSB K991R, respectively. However, cells that stably expressed CSB K991R proved difficult to maintain in cell culture, precluding a more thorough characterization of the mutant with this approach. Indeed, in the process of establishing cell lines, we realized that a sizeable fraction of the K991R cells were multi-nucleated and had an aberrant cellular morphology compared not only to WT, but also to CSB−/− cells (Supplementary Figure S2A). We also noted that a considerable fraction of the mutant cells contained micronuclei. An increased number of DNA bridges, which are possible precursors of micronucleus formation (30), were observed as well (Supplementary Figure S2B).

These initial observations prompted us to generate an inducible CSB expression system so as to minimize the deleterious effects of continuous CSB K991R expression and to allow better characterization of the mutant. Tetracycline (Tet)-inducible, stable CS1ANsv cell lines expressing N-terminally GFP/FLAG-tagged CSB WT and CSB K991R were generated, which permitted regulation of CSB expression levels with the tetracycline analogue, doxycycline (Dox) (Supplementary Figure S2C). In this cell model, little or no CSB expression occurred in the absence of doxycycline so that the cell lines could be stably maintained until the consequences of K991R mutation were to be investigated, at which point the Dox-regulated CSB gene was induced. In some instances, we found that CSB expression was ‘leaky’, possibly due to variable trace amounts of tetracycline present in the otherwise Tet-free growth media (see, for example, Supplementary Figure S2C, lane 5). Importantly, however, in contrast to the original cell lines that constitutively expressed high levels of CSB K991R, the Dox-inducible cell line could be expanded for extended periods of time without obvious growth defects, as long as Dox was withheld.

**Figure 2.**
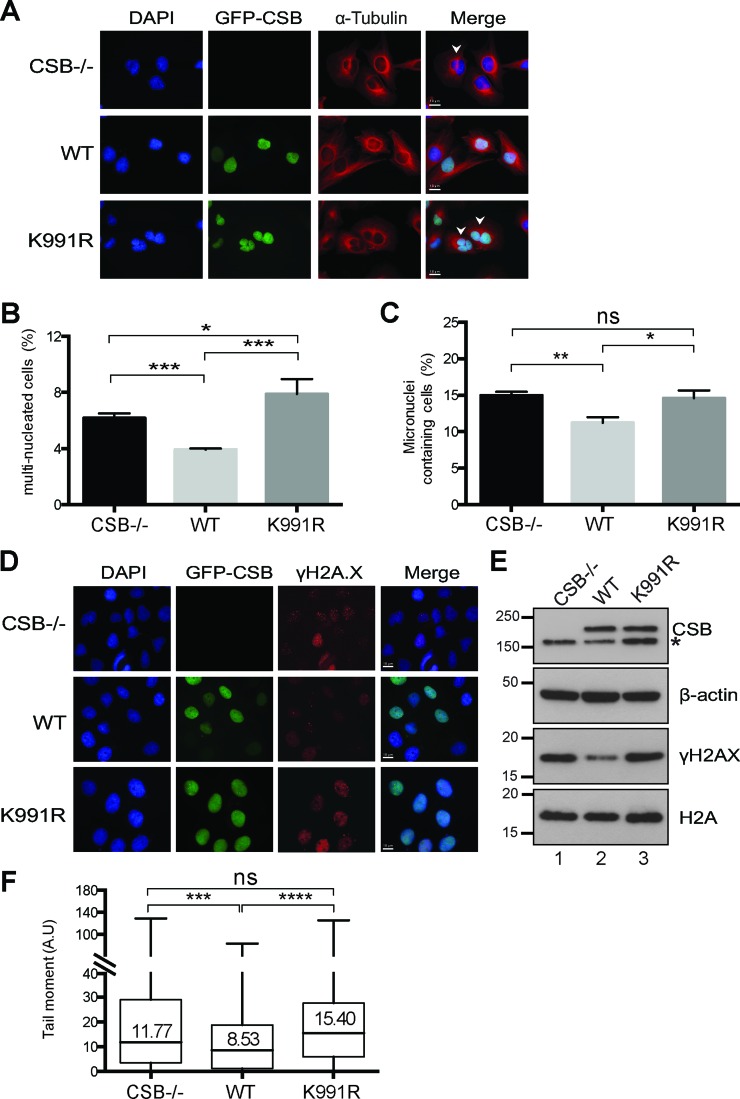
Genome instability of cells expressing CSB K991R. (**A**) Representative immunofluorescence images depicting multi-nucleated cells (white arrows in panels on right). Cytoskeleton stained for α-Tubulin and DNA stained with DAPI, while GFP-CSB was directly visualised. (**B** and **C**) Percentage of multi-nucleated (**B**), and micronuclei-containing (**C**) cells; sample size 400–500 cells. Unpaired *t*-test was used to determine statistical significant differences between the samples (*P < 0.05; **P < 0.01; ***P < 0.001; ns: non-significant). Error bars indicate the standard error of the mean from three independent experiments. (**D**) Immunofluorescence images of cells stained with γH2AX (pS139) antibody, DNA with DAPI, while GFP-CSB was directly visualized. (**E**) Western blot analysis of γH2AX (pS139) in CSB−/−, WT and CSB K991R whole cell extracts. Expression of CSB WT and CSB K991R proteins was detected by GFP antibody (asterisk indicates non-specific band), with equal loading confirmed by the β-actin and H2A controls. (**F**) Data from comet assay presented as box-whisker plots of the tail moment (A.U arbitrary units) of ∼240 individual nuclei. (ns, not significant; ***P < 0.001; ****P < 0.0001; Mann–Whitney U-test). The horizontal black bars represent the median tail moment for each dataset.

Analysis of the Dox-induced CSB cells by immunofluorescence (Figure 2A) showed that, as expected, re-expression of wild-type CSB (WT) led to a significant decrease in multi-nucleated cells and micronuclei-containing cells (Figure 2B and C, compare CSB−/− with WT). In contrast, expression of CSB K991R resulted in a marked increase of multi-nucleated cells; actually, the CSB K991R expressing cells even had a significantly greater percentage of multinucleated cells when compared to both WT and CSB−/− cells (Figure 2B). In addition, while expression of WT CSB resulted in a reduction in the number of micronuclei-containing cells, CSB K991R did not (Figure 2C).

One possible explanation for the results of the immunofluorescence experiments was that the cell cycle of CSB K991R cells is altered. FACS analysis of asynchronous cells failed to show a significant difference in the cell cycle profile of CSB K991R cells (Supplementary Figure S2D). To further investigate these observations, we performed time-lapse microscopy in order to observe and analyze the progression of individual cells through mitosis. Mitotic duration was measured as the time it takes the cells from prophase/early methaphase (morphological cell-rounding) to late anaphase/early telophase (appearance of cleavage-furrow). Interestingly, CSB K991R cells had a significant delay when undergoing mitosis compared to CSB WT and CSB−/− cells (Supplementary Figure S2E), which correlates with the multi-nucleation phenotype. In contrast, CSB−/− cells seemed to go faster through mitosis compared to CSB WT cells, which might potentially explain the elevated levels of multi-nucleation in these cells.

The higher levels of mitotic defects observed in CSB-/- and CSB K991R cells in the absence of exogenous DNA damage is indicative of increased genome instability, for which phosphorylation of histone variant H2AX at serine 139 (γH2AX) is a well-established marker (31). Immunofluorescence analysis showed that CSB-/- and CSB K991R cells indeed had pan-nuclear staining for γH2AX with some distinctive foci, whereas CSB WT cells consistently had somewhat weaker staining (Figure 2D). The difference in γH2AX levels was even more noticeable by western blot analysis (Figure 2E, panel 3, compare lanes 1 and 3 with lane 2).

The elevated level of γH2AX in CSB−/− and CSB K991R cells might indicate DNA breaks. Indeed, higher levels of DNA breaks in CSB−/− and CSB K991R cells compared to CSB WT cells were detected using an alkaline comet assay (32) (Figure 2F). This result suggests that the higher levels of γH2AX are a consequence of increased endogenous DNA damage in CSB−/− and CSB K991R cells.

Genome instability in cells expressing CSB K991R might conceivably be caused by an aberrant increase in CSB protein stability, for example, by decreasing its ubiquitylation-dependent, proteasome-mediated degradation. To investigate this possibility, cells carrying inducible CSB were grown in Dox-containing medium to establish near-normal CSB protein levels. The inducing media was then replaced with Tet-free media to cease CSB-expression, and CSB protein levels were analysed by western blot analysis. A near identical decrease in WT and CSB K991R protein levels over time was observed (Supplementary Figure S2F), indicating that mutation of the K991 ubiquitylation site does not markedly affect CSB protein stability, i.e. that it does not lead to increased proteasome-mediated degradation.

### CSB K991R ubiquitylation site mutant is proficient in TC-NER

**Figure 3.**
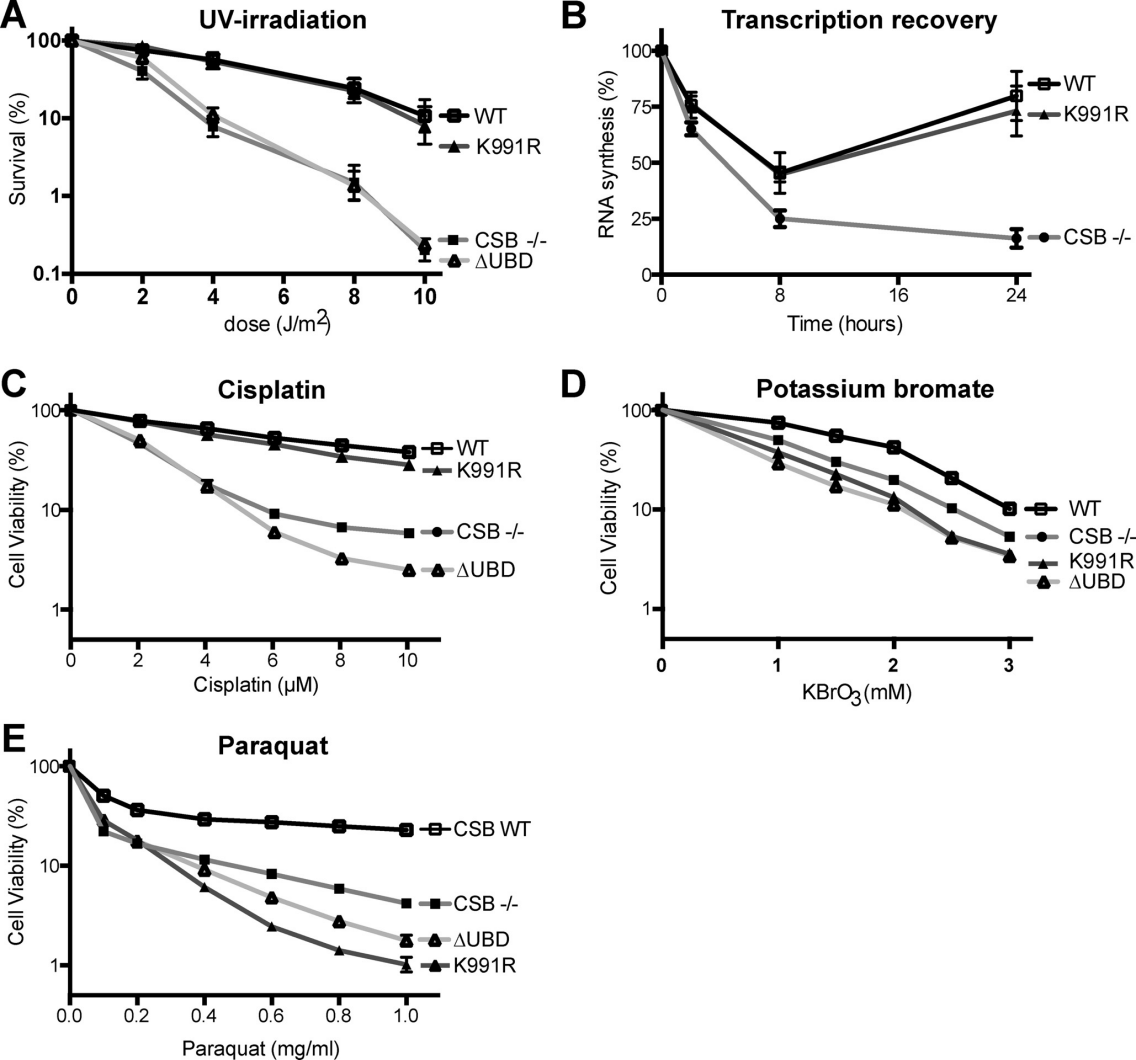
Lysine 991 is required for the response to oxidative damage, but not for TC-NER. (**A**) Clonogenic survival assay, with percentage of surviving cells (logarithmic scale) plotted against UV dose. Error bars indicate the standard error of the mean from three independent experiments. (**B**) RNA synthesis recovery after UV-irradiation (5 J/m^2^), measured as the relative incorporation of ^3^H-uridine compared to non-irradiated cells (100%). Error bars indicate the standard error of the mean from three independent experiments. (**C**–**E**) Cell viability of CSB-/-, WT and CSB K991R cells after 3 days treatment with the indicated concentrations of cisplatin (**C**), potassium bromate (**D**) and paraquat (**E**). Error bars indicate standard error of the mean from three independent experiments.

CSB is essential for TC-NER, and cells lacking CSB are sensitive to UV-irradiation (2,33). The UV sensitivity of CSB K991R cells was determined by clonogenic survival assays. As expected, CSB−/− cells were hypersensitive to UV-irradiation. Surprisingly, however, CSB K991R cells displayed a level of UV resistance similar to that of WT cells (Figure 3A). This suggests that K991 ubiquitylation is not essential for the repair of UV-induced DNA lesions by TC-NER. To provide additional evidence for this contention, we tested another cellular characteristic of CSB-deficient cells, namely failure to recover RNA synthesis after UV-irradiation (4). A drastic reduction in overall transcription levels was observed in all cell types upon UV-irradiation and, as expected, CSB−/− cells were unable to recover to normal levels (Figure 3B). In contrast, and in agreement with the data on UV-sensitivity, CSB K991R supported transcription recovery. These data confirm the proficiency in TC-NER of cells carrying K991R mutation.

### CSB K991R cells are sensitive to oxidative DNA damage

We next investigated whether CSB K991 had defects in the less well-characterized function of CSB in the cellular response to oxidative DNA damage (5,6). Sensitivity to oxidative damage was assayed using paraquat and potassium bromate (KBrO_3_). Paraquat results in the generation of superoxide radicals and hydrogen peroxide, which in turn result in oxidative DNA damage (34–36). Potassium bromate mainly generates 8-oxoguanine DNA lesions, either directly or indirectly via bromide radicals and oxides (37,38). We opted to measure cell viability after 3 days of continuous exposure to these drugs. In order to first validate these assays, cells were treated with cisplatin, which induces DNA lesions that are at least partly repaired by NER (39). Consistent with the clonogenic assays performed upon UV-irradiation, CSB−/− cells were sensitive to this cisplatin. Moreover, in agreement with previous results showing that CSB's UBD plays an important role in TC-NER (16), CSB ΔUBD cells were sensitive as well, whereas both WT and K991R cells were significantly resistant (Figure 3C).

As expected, treatment with potassium bromate and paraquat led to a dose-dependent reduction in the viability of CSB−/− cells relative to WT (Figure 3D and E). Cells expressing CSB ΔUBD were sensitive to these compounds as well. Interestingly, like ΔUBD, CSB K991R cells showed a dose-dependent decrease in viability that was similar to, or even more severe than that of CSB−/− cells. Taken together, these data indicate that K991 is dispensable for CSB's function in TC-NER, while it plays an important role in the response to oxidative DNA damage. The UBD, previously shown to be required for TC-NER (16), is required for the response to oxidative damage as well.

### CSB ubiquitylation in response to oxidative damage

**Figure 4.**
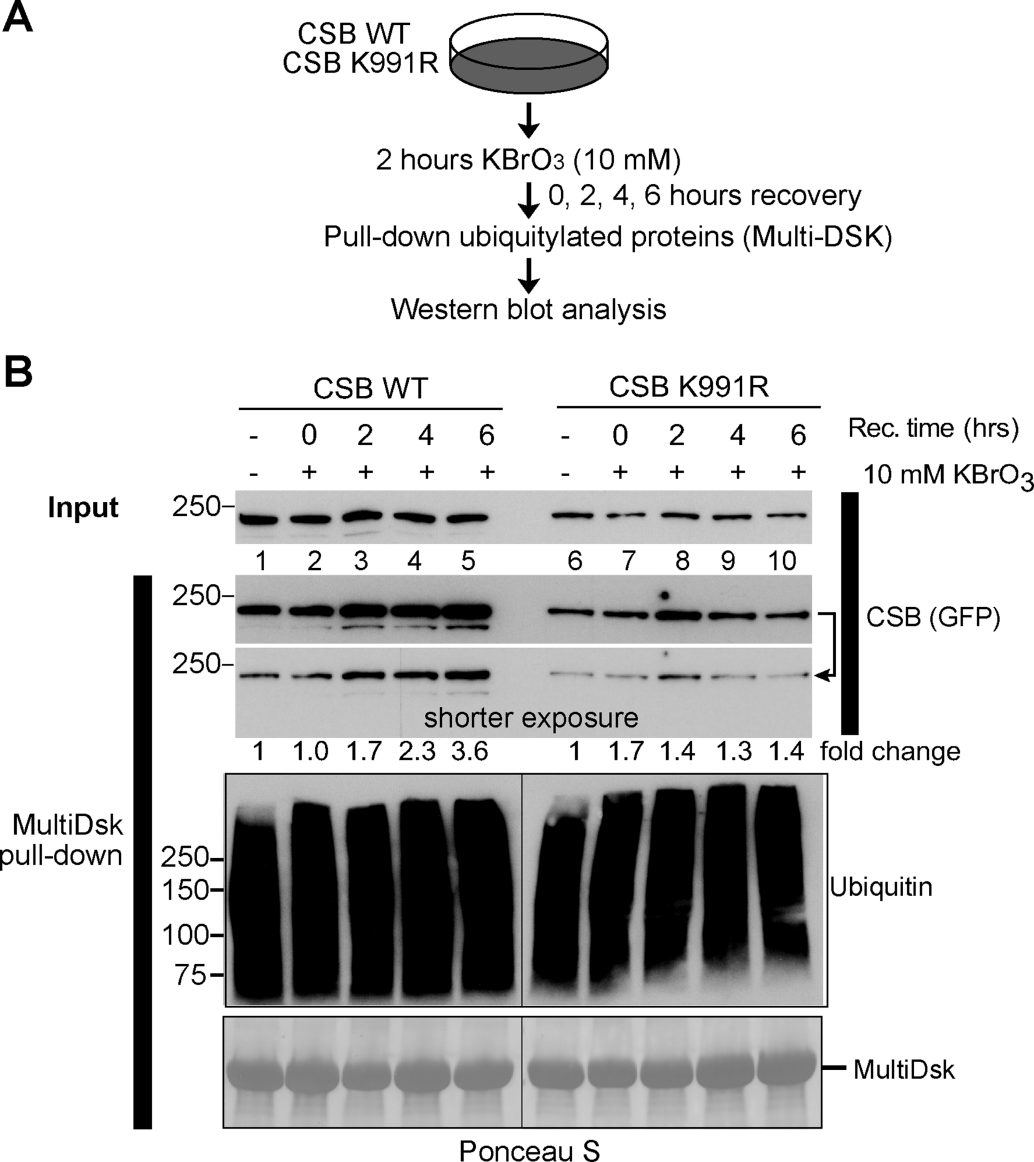
Oxidative damage induced ubiquitylation of CSB. (**A**) Outline of experimental approach. (**B**) Enrichment of ubiquitylated proteins by MultiDsk pull-down from WT and CSB K991R whole cell extracts before (−) and at different times after 2 hours of potassium bromate treatment (+). Two different exposures of the CSB blot are shown. Pull-down of ubiquitylated proteins was assessed by anti-ubiquitin immunoblot (P4D1) and the amount of MultiDsk-resin in each pull-down was visualized by Ponceau S. Note that although Dox concentration was carefully titrated so that CSB expression levels were similar, and equivalent to CSB levels in normal MRC5 cells, expression of K991R is slightly less strong than WT (<2-fold different). This does not affect the outcome of, or conclusion from, the experiment.

As described in Figure 1, we found that K991 ubiquitylation was unaffected by UV-irradiation, but whether it was induced by oxidative damage still remained to be addressed. Given that CSB can be ubiquitylated at a number of different sites, we initially attempted to raise site-specific antibodies against a branched peptide representing ubiquitylation at K991, but this approach was unsuccessful. Instead, we compared the ubiquitylation status of WT and CSB K991R (expressed at similar, near-normal levels) before and after oxidative damage by isolating ubiquitylated proteins from whole cell extracts using the ubiquitin-binding MultiDsk resin (26). WT and CSB K991R cells were treated with potassium bromate for 2 h, and ubiquitylated cellular proteins were then isolated using MultiDsk at different times during recovery (Figure 4A, schematic). The amount of ubiquitylated GFP-CSB isolated was then assessed by western blot analysis, probing with anti-GFP antibody (Figure 4B, second and third panel from top). Although some ubiquitylated CSB was isolated at all time-points, a consistent and clear increase in ubiquitylated CSB was observed after oxidative damage in WT cells, with a 2.3-fold increase after 4 hours and a 3.6-fold increase observed after 6 hours (lanes 4 and 5). Such increases were not observed in CSB K991R cells (lanes 9 and 10). We also developed a mass spectrometry approach, which allowed us to detect the ubiquitylated peptide encompassing K991 upon inducing oxidative DNA damage, which supported these data (Supplementary Figure S3).

Together, these data indicate oxidative damage-induced ubiquitylation of CSB at lysine 991.

### Oxidative-damage induced immobilisation of CSB K991R

**Figure 5.**
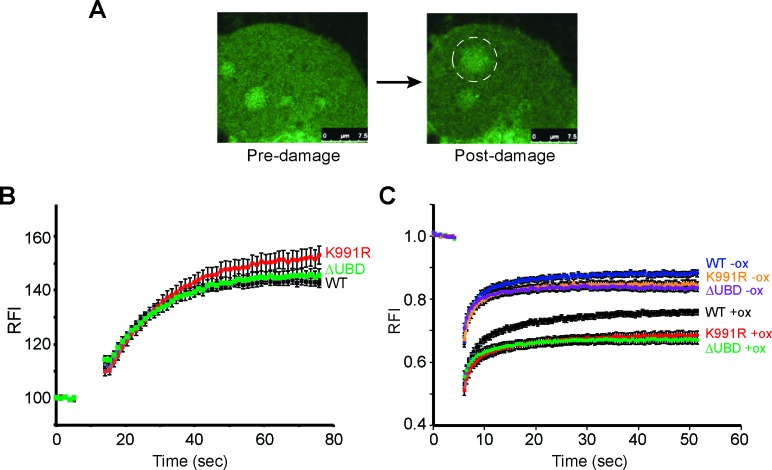
Recruitment of K991R and ΔUBD to sites of oxidative DNA damage. (**A**) Confocal microscopy images of GFP-tagged CSB K991R expressing cells before and after local induction of oxidative DNA damage (white circle). (**B**) Accumulation kinetics of relative fluorescence intensity (RFI) of WT and CSB K991R after local oxidative DNA damage (405nm with 5 μM Ro19-8022). (**C**) FRAP analysis of WT and CSB K991R in the absence and presence of oxidative DNA damage. The data of B and C represent the average fluorescence from 3 pooled, independent experiments with 15 cells each, error bars indicate the standard error of the mean of 45 cells.

Culturing cells in the presence of the photosensitizer Ro19-8022 and activating the compound using white lamp illumination allows the induction of oxidative DNA damage, predominantly 8-oxoguanine lesions (40). Using this photosensitizer and local (sub-nuclear) 405 nm laser-irradiation, it was recently shown that CSB is recruited to oxidative DNA damage (12), suggesting a direct role in contending with it. We now similarly measured accumulation of GFP-tagged CSB K991R and CSB ΔUBD at oxidative DNA damage (Figure 5A and B and Supplementary Figure S4A). Somewhat to our surprise, no major difference in the accumulation of WT and mutant proteins to the damaged site was observed (Figure 5B), suggesting that neither K991 ubiquitylation, nor the UBD are required for the initial recruitment of CSB. We therefore went on to test whether these CSB mutants might display changes in their binding dynamics at sites of oxidative DNA damage, which would suggest perturbation of the repair process. To this end, we used fluorescence recovery after photo-bleaching (FRAP) before and after localized oxidative DNA damage (12). In this assay, local oxidative damage is introduced as described above, but immediately afterwards the GFP-tagged proteins are photo-bleached using a high intensity laser pulse. The subsequent recovery of fluorescence at the bleached site provides a measure for the protein's exchange/mobility in that area of the nucleus (Supplementary Figure S4B). In the absence of damage, CSB WT and the mutant proteins had comparable fluorescence recovery kinetics, indicating similar mobility under normal conditions (Figure 5C, blue, orange and purple graphs). As expected, oxidative DNA damage significantly reduced the mobility of all CSB versions compared to the un-damaged condition (Figure 5C, black, red and green graphs). Interestingly, however, CSB K991R and ΔUBD had a marked delay in fluorescence recovery compared to CSB WT, indicating an increased residence time at the site of oxidative DNA damage (Figure 5C, compare black, with red and green graphs).

Taken together, these data indicate that CSB K991R and CSB ΔUBD are recruited normally to oxidative DNA lesions, but that they have defects in dissociation from the area of damage, indicating a similar defect in the process of dealing with the damage.

### Similar gene expression defects in cells expressing CSB K991R and CSB ΔUBD

**Figure 6.**
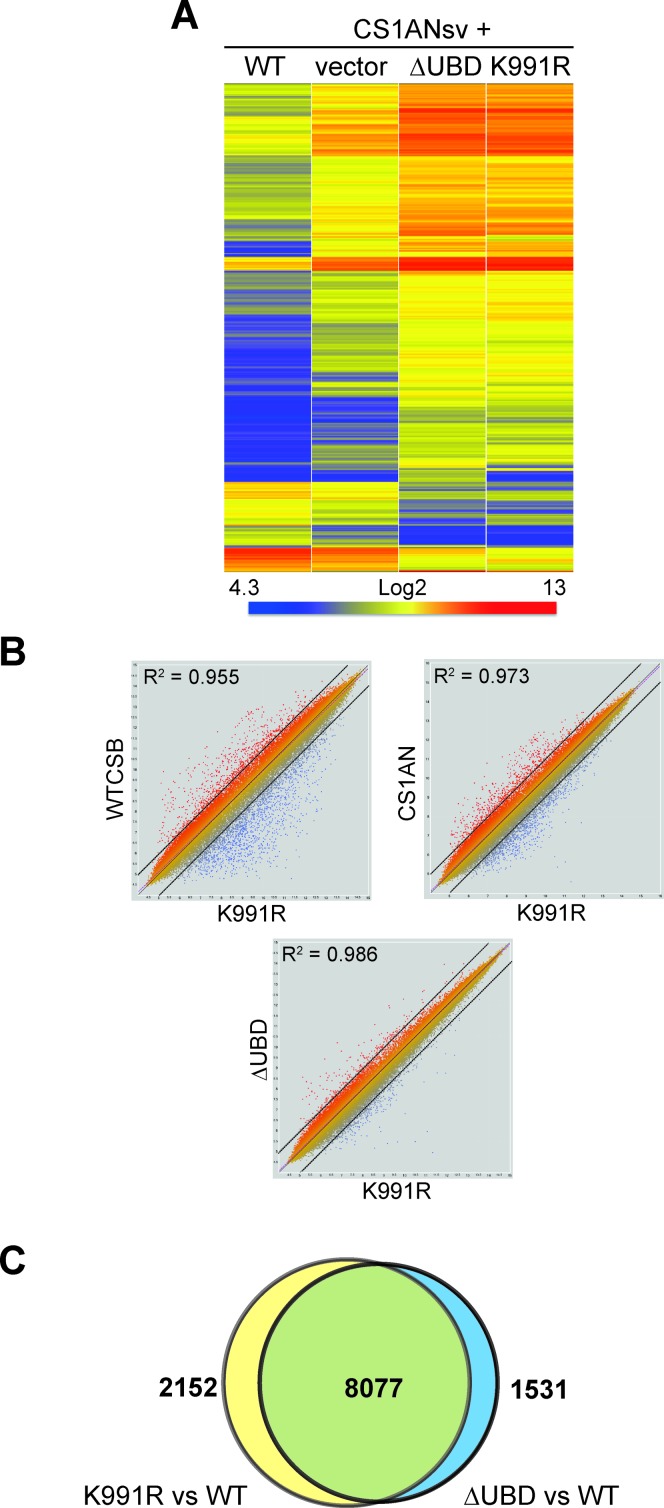
Similar gene expression defects in cells expressing K991R and ΔUBD. (**A**) Comparison of gene expression profiles in 626 genes whose expression was more than 4-fold different between WT and ΔUBD cells. Note that the patterns of gene expression were remarkably similar between ΔUBD and K991R. (**B**) Pair-wise comparisons between gene expression in K991R and WT, CS1AN (lacking CSB function altogether) and ΔUBD. (**C**) Comparison of gene expression at the gene probe level between ΔUBD and K991R cells.

The data presented above indicate a number of functional similarities between CSB K991R and CSB ΔUBD in repair of oxidative DNA damage. However, CSB plays a role in RNA polymerase II transcription as well, so we also investigated the gene expression profiles of cells expressing these mutant proteins (Figure 6). As expected from previous findings (41,42), cells lacking functional CSB altogether (vector) had a significantly different gene expression profile compared to wild type cells (WT). Interestingly, the gene expression profiles of cells expressing CSB K991R and CSB ΔUBD were remarkably similar, and differed markedly not only from WT, but also from CS1AN cells (Figure 6A; Supplementary Figure S5). Pairwise comparisons between gene expression in K991R and the other cell types underlined the striking similarity to that of ΔUBD (Figure 6B, compare the clouds of red and blue outlier genes outside stippled lines (i.e. genes at which expression is markedly different). Indeed, among 9608 gene-probes that showed differential expression between K991R cells and WT cells, 8077 (84%) also showed differential expression in ΔUBD cells (Figure 6C).

Together, these results indicate a surprisingly close functional connection between ubiquitylation at K991 and the ubiquitin-binding domain of CSB.

## DISCUSSION

Cockayne Syndrome B (CSB) is involved in RNA polymerase II transcription, transcription-coupled nucleotide excision repair (TC-NER) and repair of oxidative DNA damage, but whether and how its activity in these distinct cellular processes is determined and regulated has remained unclear. Here we show that normal function of CSB in transcription and repair of oxidative DNA damage, but not in repair of bulky DNA lesions, requires site-specific ubiquitylation at lysine 991. Remarkably, conservative mutation of this residue to arginine negatively affects genome stability, even in the absence of exogenously applied DNA damage. Moreover, cells expressing a version of CSB lacking the ubiquitin-binding domain (UBD) show defects in transcription and repair of oxidative damage as well. As a matter of fact, the defect in transcription, as measured by the gene expression profile, is remarkably similar between cells expressing CSB K991R and CSB ΔUBD, suggesting a tight functional coupling between the two.

Although the role of CSB in TC-NER is well documented (15,43), the nature of its involvement in repair of oxidative damage is much less well understood. Our results strongly support the idea that CSB's function in NER and oxidative damage repair are separable. Indeed, to our knowledge, the K991R mutation represents the first separation-of-function mutation in CSB, and it should thus be helpful for teasing apart not only the multiple functions of CSB in DNA repair, but also the contribution of these processes to a variety of cellular and organismal phenotypes resulting from CSB mutation. For example, it would be of interest to characterize the effect of such mutation in the mouse models presently used to study processes such as aging or Cockayne syndrome (44–46). Interestingly, a recent study showed that cells lacking CSB or the TC-NER factor UVSSA are defective in TC-NER of CPDs and 8-oxoG lesions. However, whereas CSB-deficient cells are sensitive to oxidative damage, UVSSA-deficient cells are not (13). Although other possibilities cannot be ruled out, this could indicate that it is normal transcriptional bypass of oxidative lesions that requires CSB, while such bypass does not require UVSSA.

Ubiquitylation of CSB at lysine 991 is important for repair of oxidative DNA damage, which might, in turn, underlie the genomic instability observed in cells expressing CSB K991R even in the absence of exogenously induced DNA damage: CSB is required for contending with DNA damage arising as a consequence of, for example, reactive oxygen species being generated as part of normal cell metabolism. We note that the K991R mutation even gave rise to phenotypes that were in some cases significantly more severe that those of cells lacking CSB function altogether. This might be at least partially explained by the finding that CSB K991R can be recruited to oxidative DNA damage, but does not function correctly there. Recruitment of defective CSB might in turn block alternative pathways contending with oxidative DNA damage.

What might be the molecular consequence of CSB ubiquitylation at K991? Unfortunately, the nature of CSB's involvement in repair of oxidative damage is largely unknown; it is thus unclear exactly why and how CSB is required for such repair. Our data show that K991 ubiquitylation (and the UBD) is dispensable for recruitment of CSB to oxidative DNA damage, but that its retention there is prolonged. This apparent ubiquitylation-dependent kinetic control of the repair reaction might be analogous to the case with the general genome (GG)-NER initiating protein XPC, which recognizes sites of DNA damage, but is displaced before actual repair takes place. Indeed, although the precise molecular mechanism also in this case remains to be determined, it was recently shown that ubiquitylation controls the release of XPC from the repair recognition complex to make space for the assembly of downstream repair factors and thereby facilitating efficient damage removal (47). CSB is likely to be involved in displacing the lesion-stalled RNA polymerase II, or nucleosomes, for example to provide working space for the DNA repair factors. Indeed, previous data showed that the recruitment of CSB to oxidative damage is transcription-dependent (12). Whether oxidative DNA damage directly causes stalling of RNA polymerases has been a matter of debate (48–52), but CSB is capable of stimulating transcript elongation through DNA templates containing 8‐oxoguanine lesions *in vitro* (48). It is thus conceivable that oxidative DNA damage or BER-intermediates cause problems for RNAPII transcript elongation and that CSB is recruited to facilitate remodeling of the transcription machinery, or chromatin, and thus allow subsequent progression of the BER reaction. Alternatively, or additionally, it might be involved in restarting transcription after such repair has taken place.In apparent agreement with these ideas, K991R mutation also affects transcription on a genome-wide scale. Importantly in this connection, very similar gene expression defects are observed in cells expressing CSB lacking the UBD and those carrying K991R mutation. This, combined with the similar effect of these mutations in other assays, suggests that ubiquitylation at K991 and the function of the UBD are very tightly coupled. Interestingly, however, whereas K991R mutation does not affect NER, the UBD is required for it (16). Uncovering the mechanism underlying the differential effects of perturbing CSB ubiquitylation and the UBD will have to await the development of cell assays to characterize the molecular mechanism of CSB-dependent BER.

## Supplementary Material

Supplementary Data

SUPPLEMENTARY DATA
