# Supplementary material for: A ubiquitylation site in Cockayne syndrome B required for repair of oxidative DNA damage, but not for transcription-coupled nucleotide excision repair
Source: Nucleic Acids Res. 2016 Apr 7;44(11):5246–55. doi: 10.1093/nar/gkw216 (PMC4914099; doi:10.1093/nar/gkw216)
Supplement: Supplementary Data [file gkw216_Supplementary_Data.zip › nar-03421-d-2015-File008.pdf]

A

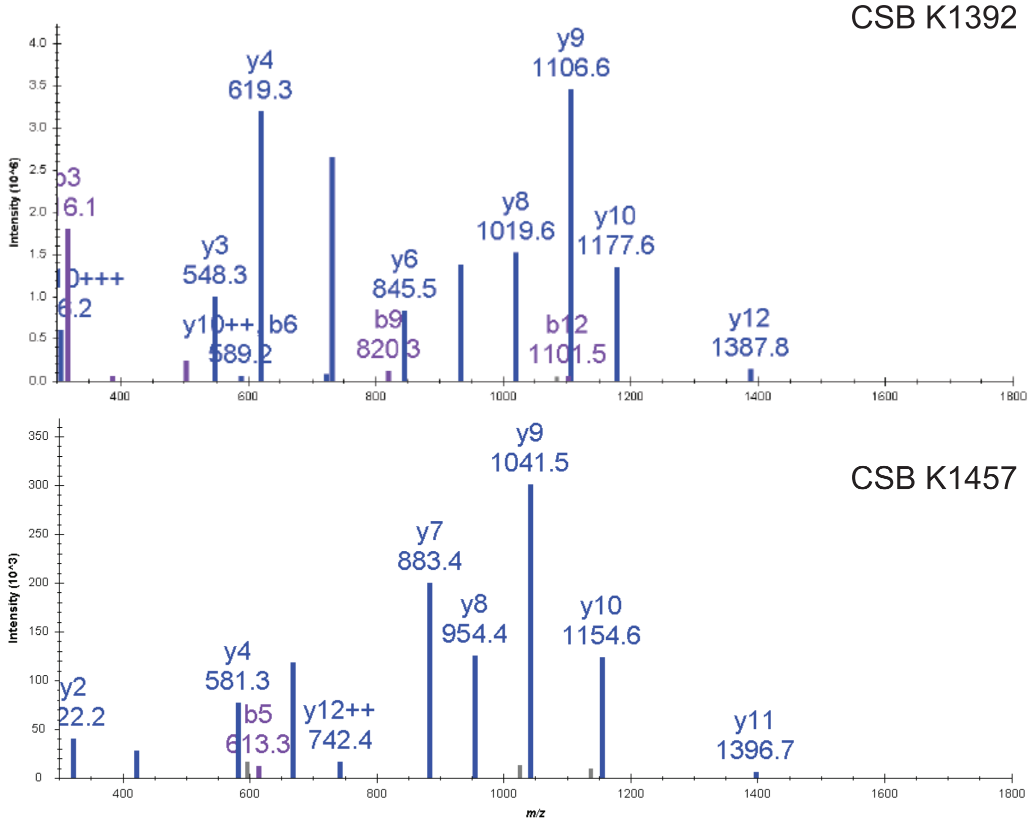

B

```
K1392
Human        PEHFSGRAEDADSSSGPLASSSLLAKMRARNHLILPERLESES 1409
Mouse        PEHFSGKEDGA-SVSGAPSSSSLLARMRARNHMILPERLESDS 1396
Dog          SEHFSGKVEDAESSPGALPSSSLLAKMRARNHLILPQRLESEN 1401
Chicken      NGHFDGKSETGESSSSILDSSSLLAKMRARNHLILPQRTGNEG 1409
Opposum      QTHFSGKIEGGESSSGALASSSLLAKMRTRNHLILPQRIENEN 1407
Zebrafish    AAHFSGEGAEDE--SGSLSSSSLLARMRARNHLKKPQSQEDEE 1305
Puffer       SAHFSGEGPEGEAEGAELSSSTLLAKMRVRNYLSAPPSQRDGE 1262
                **.*.         .    **:***:**.**::  *     .
K1457
Human        QAHTDGQASTREILQEFESKLSASQSCVFRELLRNLCTFHRTS 1480
Mouse        QAQVDGQASTQEILQEFESKLSVAQSCVFRELLRNLCNFHRTP 1468
Dog          QARVDGQASTQEILQEFESKLSASQSCVFRQLLRNLCTFHRTS 1472
Chicken      QARVDGEASTQEILQEFESKLPAAQSCVFRELLRNLCSFHRNP 1482
Opposum      QARIDGQASTQEILQEFESKLSTSQSCVFRELLRNLCTFHRGL 1481
Zebrafish    QAQVDGQASTKEILEYFTPRLTSTQMPVFRELLNNICEFHRLP 1377
Puffer       QAGVDGQATTQELLGYFRPRLSQQQAPVFRELLRSICHFHRTA 1335
             **   **:*:*:*:*  *   :*    *   ***:**..:* ***
```

C

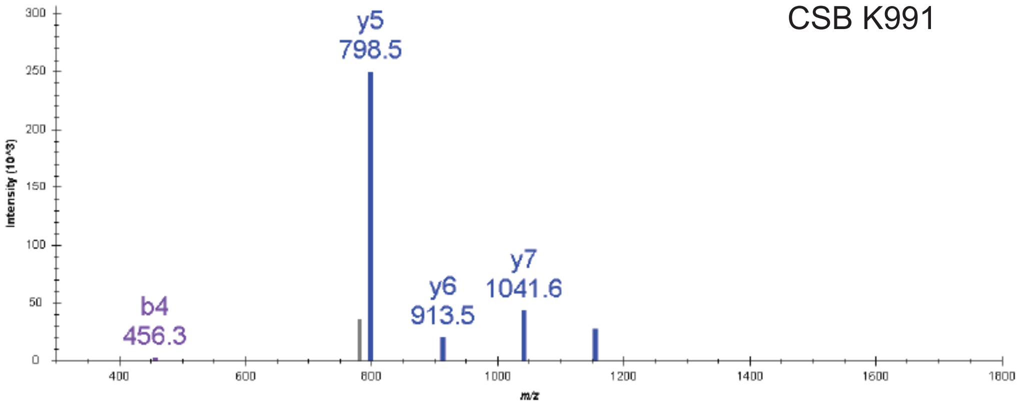

Supplementary Figure S1

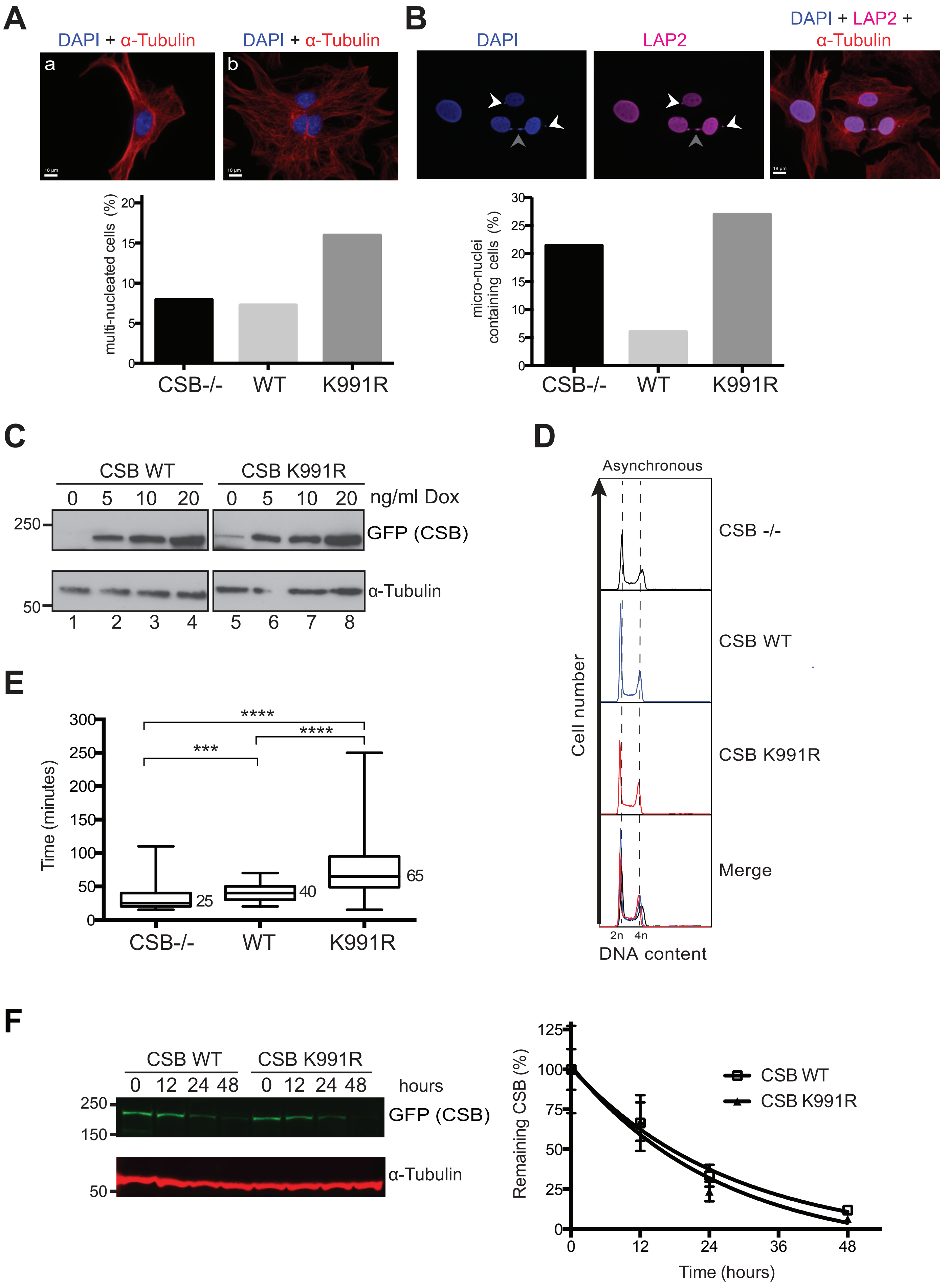

Supplementary Figure S2

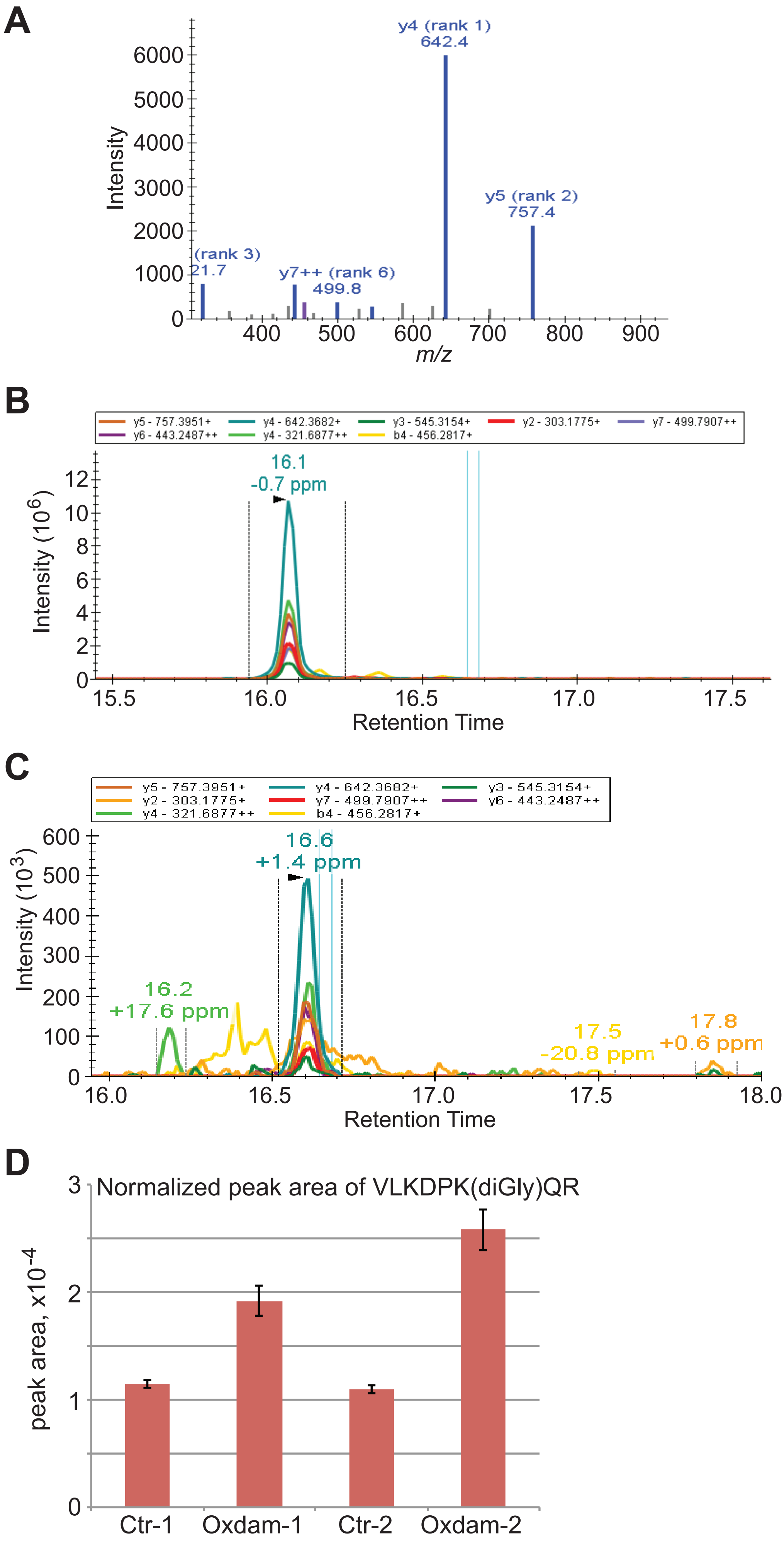

Supplementary Figure S3

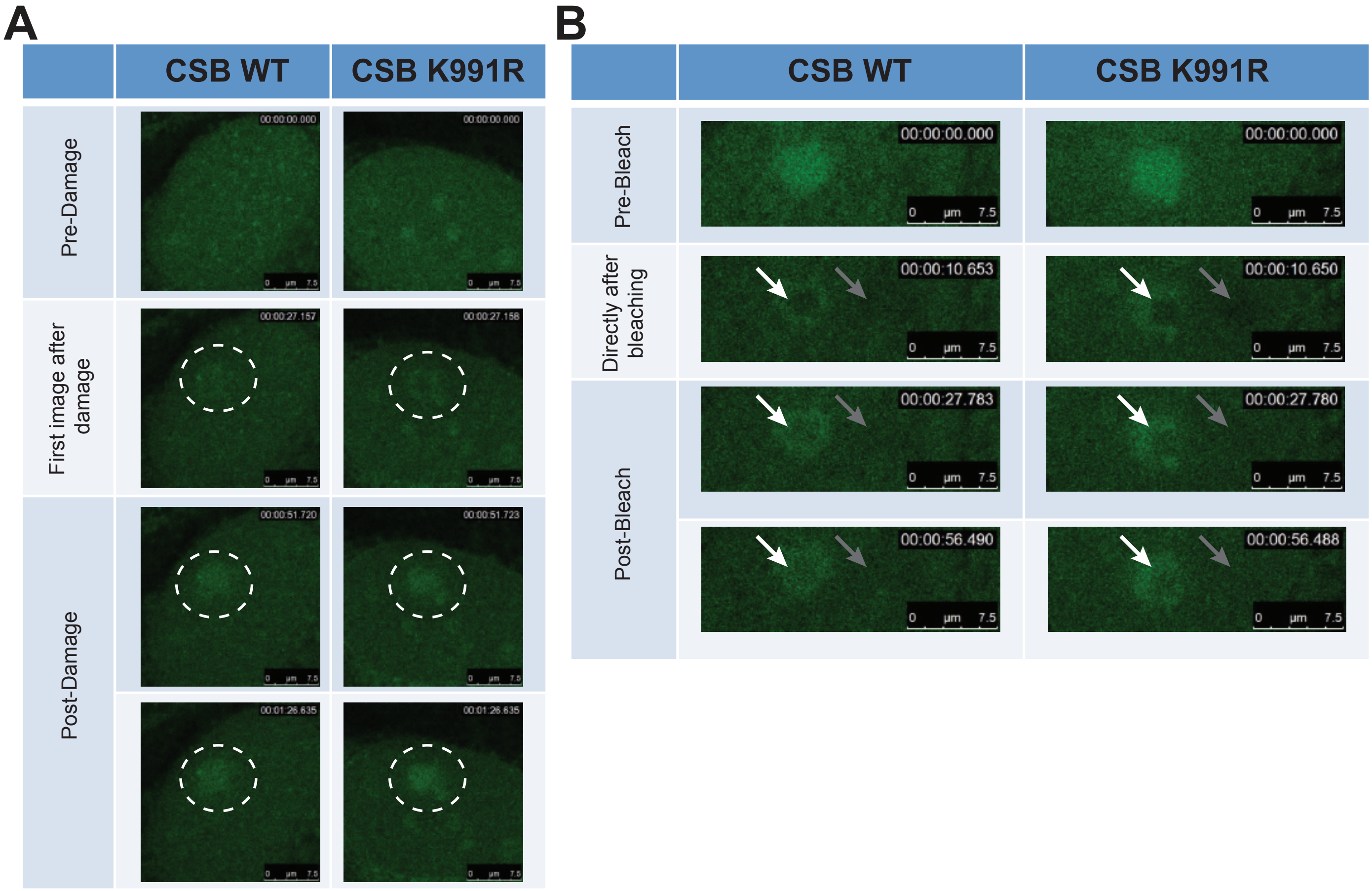

Supplementary Figure S4

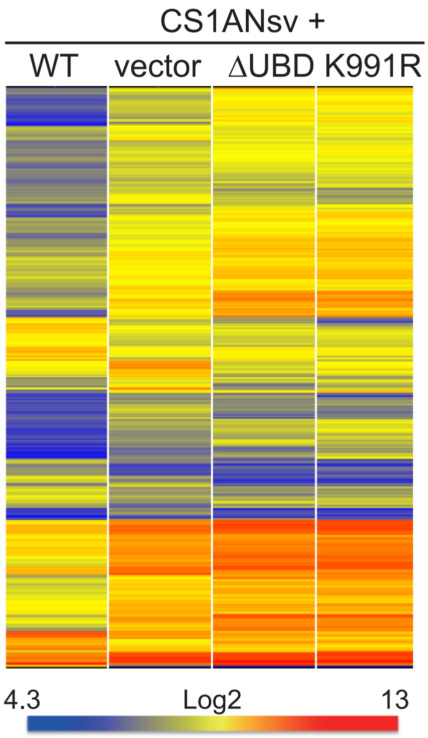

Supplementary Figure S5

Supplementary Figure Legends

Supplementary Figure S1. Identification of novel CSB ubiquitylation sites.

A. CSB ubiquitination sites were identified by mass spectrometry following protein digestion and enrichment of diGly containing peptides. A range of ubiquitination sites were detected including lysine 1392 (K1392) and lysine 1457 (K1457). The sites were identified with MaxQuant where the FDR was restricted to 1% on peptide and site level. The MS2 fragmentation spectra of the corresponding peptides (AEDADSSSGPLASSSLLAK(diGly)MR and EILQEFESK(diGly)LSASQSCVFR) are presented and all matching y and b ions are annotated with their measured m/z (mass to charge). **B.** Multiple sequence alignments of CSB vertebrate orthologues depicting the sequence around lysine 1392 and lysine 1457 (highlighted, red arrow). Note that this either arginine (which cannot be ubiquitylated) or lysine are found at these positions. **C.** MS2 fragmentation spectra of peptide VLKDPK(diGly)QRR containing lysine 991 (K991) ubiquitination site**.** All matching y and b ions are annotated with their measured m/z (mass to charge).

Supplementary Figure S2. Initial cellular phenotypes CSB K991R expression and protein stability. A, Upper. Representative immunofluorescence images of mono-nucleated CSB WT (a) and multi-nucleated CSB K991R cells (b). Cytoskeleton was stained for α-tubulin and DNA stained with DAPI. Lower, percentage of multi-nucleated cells present in randomly counted sample size of approximately 250 cells. **B.** Representative immunofluorescence image of CSB K991R cells containing

micronuclei (white arrowheads) and DNA bridges (grey arrowheads). DNA was stained with DAPI, nuclear membrane was stained for the LAP2, while the cytoskeleton was stained for α-tubulin. Percentage of micronuclei containing cells present in randomly counted sample size of approximately 350 cells. For both A and B quantification: as these experiments were later followed up using the inducible cell line, only the single experimental data sets that was generated with the initial cell line are shown. **C.** Western blot analysis of whole cell extracts of Tet-inducible cell lines; CSB WT and CSB K991R expression after induction with increasing amounts of doxycycline. CSB was probed with anti-GFP antibody and α-tubulin was used as loading control. **D, Upper.** Quantitative western blot analysis of CSB WT and CSB K991R protein levels after repression of doxycycline-induced CSB expression over a 48 hours time period. CSB was probed with anti-GFP antibody and α-tubulin was used as loading control. **Lower.** Graph representing the turnover kinetics of CSB WT and CSB K991R proteins calculated from immunoblots with the GFP-CSB signal normalised to α-tubulin. Error bars indicate the standard error of the mean from three independent experiments.

Supplementary Figure S3. Identification and confirmation of K991 ubiquitylation by mass spectrometry. A. The K991-containing CSB peptide VLKDPK(diGly)QR was identified using an IP-Mass Spectrometry approach with a Mascot score of 25. A targeted proteomics assay (parallel reaction monitoring) was subsequently set up to confirm the identity and presence of this peptide. **B.** Elution profile of a synthetic peptide with the sequence VLKDPK(diGly)QR; the predicted

transition (y and b ions) from the spectral library are continuously monitored throughout the run using Skyline software. **C.** Confirmation of the presence of the VLKDPK(diGly)QR peptide in the CSB-IP after oxidative damage showing good retention time reproducibility (± 1 min). **D.** Comparison between control (Ctr) and sample treated with 10 mM potassium bromate (Oxdam). Each sample was analysed in two technical replicates. Peak areas of all measured peptides were integrated using Skyline. The area of the VLKDPK(diGly)QR peptide was normalised to total protein using three unmodified CSB peptides. Peptides chosen for normalisation (VLLFSQSR, LLTAGTIEEK and VGGLGVNLTGANR) had high abundance and did not contain any cysteines or methionines. Standard errors reflecting the variation between normalisation procedures are displayed.

Supplementary Figure 4. CSB immobilisation in response to oxidative DNA damage. **A.** Confocal microscopy images of cells expressing GFP-tagged CSB WT and CSB K991R before and after local induction of oxidative DNA damage (white dotted circles). **B.** Confocal microscopy images of GFP-tagged CSB WT and CSB K991R depicting the fluorescence recovery after photobleaching after the local induction of oxidative DNA damage (white arrows) or undamaged control area (grey arrows).

Supplementary Figure 5. Similar gene expression defects in cells expressing K991R and ∆UBD. Comparison of gene expression profiles in 930 genes whose

expression was more than 2-fold different between WT and ΔUBD cells. Note that the patterns of gene expression were remarkably similar between ΔUBD and K991R.
