# Supplementary material for: A ubiquitylation site in Cockayne syndrome B required for repair of oxidative DNA damage, but not for transcription-coupled nucleotide excision repair
Source: Nucleic Acids Res. 2016 Apr 7;44(11):5246–55. doi: 10.1093/nar/gkw216 (PMC4914099; doi:10.1093/nar/gkw216)
Supplement: SUPPLEMENTARY DATA [file supp_44_11_5246__index.html]

A ubiquitylation site in Cockayne syndrome B required for repair of oxidative DNA damage, but not for transcription-coupled nucleotide excision repair — A ubiquitylation site in Cockayne syndrome B required for repair of oxidative DNA damage, but not for transcription-coupled nucleotide excision repair — A ubiquitylation site in Cockayne syndrome B required for repair of oxidative DNA damage, but not for transcription-coupled nucleotide excision repair — SUPPLEMENTARY DATA 

# A ubiquitylation site in Cockayne syndrome B required for repair of oxidative DNA damage, but not for transcription-coupled nucleotide excision repair

## SUPPLEMENTARY DATA

- SUPPLEMENTARY DATA
